# Characteristics and determinants of clinical symptoms in radiographic lumbar spinal stenosis in a tertiary health care centre in sub-Saharan Africa

**DOI:** 10.1186/s12891-017-1844-2

**Published:** 2017-11-28

**Authors:** Marie Doualla-Bija, Mbeng Ashu Takang, Emmanuella Mankaa, Jude Moutchia, Pierre Ongolo-Zogo, Henry Luma-Namme

**Affiliations:** 1 Faculty of medicine and Biomedical Sciences, University of Yaounde I, Yaoundé, Cameroon; 2 General Hospital Douala-Cameroon, P.O. Box 4856, Douala, Africa Cameroon

**Keywords:** Lumbar spinal stenosis, Symptoms, Computed tomography, Africa

## Abstract

**Background:**

Lumbar spinal stenosis (LSS) refers to narrowing of the lumbar central spinal canal, lateral recess, and/or neuro-foramina. Radiographic LSS plays an important role in clinical LSS but is not solely accountable for the presence of symptoms. We sought to characterise clinical LSS and to determine factors associated with presence of symptoms of LSS in patients with radiographic LSS in a sub Saharan Africa setting.

**Methods:**

After prior ethical clearance, a case control study was done in a tertiary hospital in Douala-Cameroon, including 105 patients with radiographic LSS: 57 with symptoms of LSS (cases) and 58 with no symptoms (controls). Spinal stenosis was assessed using computed tomography (CT) scans. Data were analysed using SPSS version 23.

**Results:**

The mean age of our study participants was 53.4 ± 13.1 years. The mean age of onset of symptoms of LSS was 50.3 ± 11.6 years and the most common symptoms were Low back pain (100.0%), radicular symptoms (98.2%) and neurogenic claudication (98.2%). Obesity (*p* < 0.001) and a high waist circumference (*p* = 0.002) were significantly associated with presence of LSS symptoms in persons with radiographic LSS. After adjusting for body mass index, a positive family history of low back pain (*p* = 0.004), vertebra lesion at L2 (*p* = 0.034), L3 (*p* = 0.002), L4 (*p* = 0.025) and multiple (*p* = 0.008) levels, degenerative disc protrusion (*p* = 0.044), disc lesion at L3-L4 (*p* = 0.001), L4-L5 (*p* = 0.011) and multiple (*p* = 0.046) levels were significantly associated with presence of symptoms of LSS in persons with radiographic LSS.

**Conclusion:**

Characteristics of clinical LSS have been described in this sub-Saharan Africa population. Obesity, a high waist circumference and a positive family history of low back pain are significantly associated with presence of symptoms of LSS in persons with radiographic LSS.

## Background

Lumbar spinal stenosis (LSS) refers to narrowing of the lumbar central spinal canal, lateral recess, and/or neuro-foramina [1, 2]. The resultant disproportion between the size of neural elements and available space leads to encroachment of neural and vascular structures. It is most commonly caused by degenerative changes either in the disc, facet joints, ligaments or vertebrae body [3]. LSS is most common in individuals in the 6th decade of life and above, and its prevalence increases with age [4]. Approximately 1 per 1000 persons older than 65 years and about 5 of every 1000 persons older than 50 years in USA have symptoms of LSS [5]. Ishimoto et al. [6] reported prevalence of moderate or severe central stenosis of 64.0% in patients in their 50s and 93.1% in those in their 80s.In Africa, studies have shown that LSS occurs at earlier ages [7] and is more prevalent [8] compared to that in the western world. These discrepancies have been attributed to the tough nature of tasks carried out by Africans daily [8]. This attribution is supported by the fact that weight bearing activities decrease spinal canal dimensions [9]. LSS remains a major cause of morbidity, disability and lost productivity [10]. Increase in global life expectancy [11] and a projected increase of the worldwide percentage of older people (>65 years) from 11.7% in 2013 to 21.1% by 2050 [12] means that global prevalence of LSS may increase steadily, thus gaining increased attention. The rate of complex fusion procedures for LSS in the US increased 15-fold from 2002 to 2007 [13] and LSS has now become the most common indication for spine surgery in Sweden [14].

Clinical LSS (symptomatic LSS) is diagnosed by the presence of neurogenic claudication, radicular symptoms, or both, with or without low back pain, in the presence of radiographic LSS [2, 15]. Biometric parameters are used to demonstrate narrowing of lumbar central spinal canal, lateral recess, and/or neuro-foramina in radiographic LSS [16]. Different imaging techniques (x-ray, myelography, computed tomography [CT], and magnetic resonance imaging [MRI]) are used in the radiographic evaluation of LSS [17]. MRI is most commonly used and there is consensus it yields the best soft tissue contrast [17]. However, there is still no consensus on the set of features which define radiographic LSS [18, 19]. Most studies use criteria set by Verbiest [20]; diameter of 10–12 mm for relative spinal stenosis and <10 mm for absolute spinal stenosis [16].

Based on the pathophysiology of LSS, there is an expected correlation between radiographic features and symptoms of LSS. There has been conflicting results on the association between radiographic findings of stenosis and symptoms of LSS [6, 21–23]. Ishimoto et al. showed that the prevalence of clinical symptoms increased with increasing severity of radiographic LSS [6]. Hurri et al. in a 12-year follow-up period showed an association between Oswestry Disability Index and degree of stenosis [23]. However, many other studies found no correlation between the degree of radiographic stenosis and clinical symptoms [24–30]. In a cross-sectional study of adults in Japan, prevalence of moderate or severe radiographic LSS (76.5%) was much higher than prevalence of symptomatic LSS (9.3%) in the same group of participants [21]. Some individuals with very mild radiographic stenosis present with very severe disabling symptoms while some individuals with severe radiographic stenosis are asymptomatic [31]. The discrepancy between radiographic LSS and symptoms of LSS is further compounded by the equivocal response by patients to decompressive surgery [32, 33]. In Africans, very little is known about the correlation between radiographic and clinical LSS.

The aims of this study were to describe the characteristics of clinical LSS and to determine factors associated with presence of symptoms of LSS in patients with radiographic LSS in a sub-Saharan Africa setting.

## Methods

### Study participants

We carried out a case control study in the Radiology Unit of the Douala General Hospital, Cameroon, from December 2014 to April 2015. After ethical clearance from an institutional review committee, we targeted patients aged 21 years and above, undergoing a lumbar spine or abdomino-pelvic CT scan during the study period. We excluded persons with a history of spine surgery and persons with an ankle-brachial index ≤0.90.

**Fig. 1:**
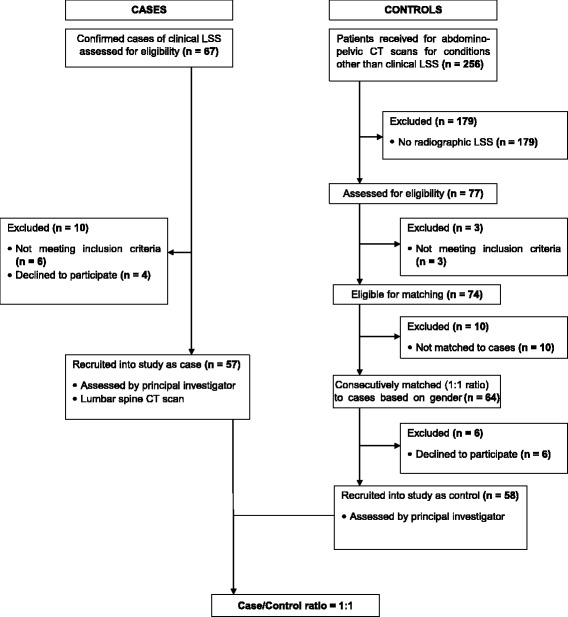
Flow chart of study participants

Cases included patients with confirmed clinical LSS (clinical syndrome of neurogenic claudication, radicular symptoms, or both, with or without low back pain and presence of radiographic LSS). They were consecutively enrolled into the study after consent was obtained. Controls were recruited from patients referred to our radiology unit for abdomino-pelvic CT scans for other conditions, and included patients with no clinical evidence of LSS who had radiographic LSS on supplementary spine CT scan analysis. They were consecutively matched to cases in a ratio of 1:1 based on gender, and after consent was obtained, they were enrolled into the study Fig. 1.

### Clinical assessment

All study participants were assessed by the principal investigator and findings were filled into a pretested data collection sheets. Cases were assessed within 2 h before lumbar CT scan was done and data collected included demographic characteristics, history of symptoms and physical findings. Controls were assessed after supplementary analysis of their abdomino-pelvic CT scans showed radiographic LSS. Assessment was done when these patients presented for their CT scan results within 3 days; data collected included demographic characteristics and anthropometric measurements.

Occupation was classified using the international standard classification of occupations (ISCO-08) [34]. Pain was assessed using a linear visual analogue scale (VAS) ranging from zero (no pain) to 10 (unbearable pain) [35]. Height and weight were measured and body mass index (BMI) computed; obesity was considered as BMI ≥ 30.0 kg/m^2^, overweight as 25.0 ≤ BMI < 30 kg/m^2^, normal weight as a BMI 18.5 ≤ BMI < 25 kg/m^2^ and underweight as BMI < 18.5 kg/m^2^ [36]. Waist circumference (WC) was measured with the use of a measuring tape according to World Health Organisation guidelines; WC was considered high if >102 cm in men, > 88 cm in women [36]. Sitting height measured the distance from the highest point of the head to the flat surface of a chair [37]. The participants were made to sit erect, looking straight ahead, both feet on the floor, knees put together, the lower back and shoulders against the wall. The relative sitting height was computed as (sitting height (cm) × 100)/standing height (cm) [38].

### Radiographic assessment

CT scan (8-slice Hitachi® and a 2-slice General Electronics®) imaging was used to assess LSS. In order to minimise investigator bias, CT scan images of participants recruited into the study were analysed by a radiologist blinded to the group of the participant. The items assessed included: type(s) (bony, joint or disc) and level(s) of vertebral lesion, type(s) and level(s) of disc disease, and ligamentum flavum hypertrophy. Biometric measurements of the lumbar vertebrae were automated generated CT measurements. The criteria used to define stenosis were as follows:

i.) Central canal stenosis: presence of any of the following; antero-posterior diameter of central canal <10 mm [39, 40], transverse (inter-pedicular) diameter of central canal <16 mm [40, 41] or cross-sectional area of dural sac < 100 mm^2^ [40, 42].
ii.) Lateral stenosis: presence of any of the following; depth of lateral recess ≤3 mm [40, 43], height of lateral recess ≤2 mm [40, 44] or angle of lateral recess <30^0^ [40, 45].
iii.) Foraminal stenosis; antero-posterior diameter of the foramen ≤3 mm [40, 46].

### Statistical analysis

Statistical significant difference between proportions was assessed using the Pearson’s chi-squared test, and between means using independent samples student’s *t*-test. Bivariate analysis was done using logistic regression to estimate the odds ratio of having symptoms of LSS. BMI class and waist circumference class were significantly different between cases and controls (Table 1), and were correlated on Cramer’s V (ϕ_c_: 0.55, *p* value: 0.001). Therefore, on multivariate analysis using logistic regression, in order to deal with multicollinearity, we adjusted the odds ratio of having symptoms of LSS for BMI class only. Analysis was done using Statistical Package for Social Sciences (SPSS), version 23 Inc., Chicago, Illinois, USA. Statistical significance was set at α ≤ 0.05.

**Table 1:** Characteristics of study participants

Parameter	Cases (*N* = 57)	Controls (*N* = 58)	Total (*N* = 115)	*p* - value
Age (years), mean ± SD	55.23 ± 12.89	51.52 ± 13.09	53.36 ± 13.07	0.128
Age strata, n (%)				0.285
< 40	6 (10.5)	10 (17.2)	16 (13.9)	
40–49	12 (21.1)	12 (20.7)	24 (20.9)	
50–59	17 (29.8)	19 (32.8)	36 (31.3)	
60–69	12 (21.1)	14 (24.1)	26 (22.6)	
70–79	10 (17.5)	3 (5.2)	13 (11.3)	
Female, n (%)	31 (54.39)	32 (55.17)	63 (54.78)	0.932
Occupation				0.441
Managers	1 (1.8)	1 (1.7)	2 (1.7)	
Professionals	7 (12.3)	9 (15.5)	16 (13.9)	
Clerical support workers	8 (14.0)	7 (12.1)	15 (13.0)	
Service and sales workers	9 (15.8)	11 (19.0)	20 (17.4)	
Skilled agricultural, forestry and fishery	18 (31.6)	9 (15.5)	27 (23.5)	
Craft and related trades workers	7 (12.3)	7 (12.1)	14 (12.2)	
Elementary occupations	7 (12.3)	14 (24.1)	21 (18.3)	
Mean Weight (kg), mean ± SD	87.46 ± 20.95	74.05 ± 12.76	80.71 ± 18.51	<0.001
Mean Height (m), mean ± SD	1.66 ± 0.09	1.69 ± 0.09	1.68 ± 0.09	0.054
Mean BMI, (kg/m^2^), mean ± SD	31.93 ± 8.67	25.80 ± 4.31	28.84 ± 7.47	<0.001
BMI Class, n (%)				<0.001
Normal	10 (17.5)	26 (44.8)	36 (31.3)	
Overweight	12 (21.1)	27 (46.6)	39 (33.9)	
Obese	35 (61.4)	5 (8.6)	40 (34.8)	
Waist circumference (cm), mean ± SD	102.19 ± 14.54	90.19 ± 9.08	96.14 ± 13.47	<0.001
High waist circumference, n (%)	37 (64.9)	25 (43.1)	62 (53.9)	0.019
Sitting height (cm), mean ± SD	81.91 ± 7.3	82.69 ± 5.37	82.30 ± 6.36	0.514
Relative sitting height, mean ± SD	49.29 ± 3.26	48.84 ± 2.88	49.07 ± 3.07	0.437
Radiographic Stenosis, n (%)				0.352
Central	32 (56.1)	33 (56.9)	65 (56.5)	
Foraminal	23 (40.4)	25 (43.1)	48 (41.7)	
Lateral	2 (3.5)	0 (0.0)	2 (1.7)	
Type of vertebra lesion, n (%)				0.073
None	13 (22.8)	16 (27.6)	29 (25.2)	
IAJOH	33 (57.9)	24 (41.4)	57 (49.6)	
Osteophytes	8 (14.0)	18 (31.0)	26 (22.6)	
Listhesis	2 (3.5)	0 (0.0)	2 (1.7)	
Tumoral	1 (1.8)	0 (0.0)	1 (0.9)	
Type of disc lesion, n (%)				0.173
None	10 (17.5)	22 (37.9)		
IDD	8 (15.8)	10 (17.2)		
IDP	5 (8.8)	6 (10.3)		
IDH	5 (8.8)	5 (8.6)		
HP	2 (3.5)	2 (3.4)		
DDP	15 (26.3)	8 (13.8)		
DDH	11 (19.3)	5 (8.6)		
Disc lesion level, n (%)				
L1-L2	2 (3.5)	0 (0.0)	2 (1.7)	0.100
L2-L3	7 (12.3)	1 (1.7)	8 (7.0)	0.009
L3-L4	22 (38.6)	4 (6.9)	26 (22.6)	<0.001
L4-L5	37 (64.9)	19 (32.8)	56 (48.7)	<0.001
L5-S1	31 (54.4)	37 (63.8)	68 (59.1)	0.556
Multi-level	33 (57.9)	19 (32.8)	52 (45.2)	0.007
Ligamentum Flavum hypertrophy, n (%)	32 (56.1)	35 (60.3)	67 (58.3)	0.648

*BMI* body mass index, *IAJOH* inter apophysial joint osteoarthritis and hypertrophy, *IDD* isolated degenerative disc, *IDP* isolated disc protrusion, *IDH* isolated disc herniation, *HP* disc herniation and protrusion, *DDP* degenerative disc protrusion, *DDH* degenerative disc herniation

## Results

During the study period, we received a total of 67 cases of confirmed clinical LSS. Of these, we excluded 10 cases: 6 did not meet the inclusion criteria and 4 declined to participate. A total of 57 cases (26 males, 31 females) were recruited into the study, with a participation rate of 93.4% for cases. We had 77 cases of radiographic LSS, out of the 256 abdomino-pelvic CT scans of patients without clinical LSS evaluated. Seventy-four were eligible for matching, 64 were consecutively matched to cases and 6 declined to participate. We recruited of total 58 controls (26 males, 32 females) into the study, with a participation rate of 90.6% for controls Fig. 1.

### Baseline characteristics of study participants

The mean age of our study participants was 53.4 ± 13.1 years; 55.2 ± 12.9 years for cases and 51.5 ± 13.1 years for controls (*p* = 0.128). Sixty- five (56.5%) participants had central stenosis (56.1% cases and 56.9% controls), 48 (41.7%) had foraminal stenosis (40.4% cases and 43.1% controls) and 2 (1.7%) had lateral stenosis (3.5% cases and no controls) Table 1.

**Fig. 2:**
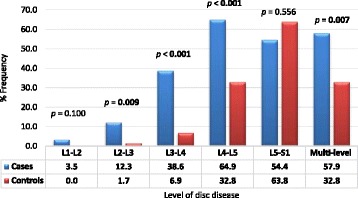
Level of disc disease in study participants

Degenerative lesions of the spine involved: 49.6% zygapophyseal joint lesions (osteoarthritis and hypertrophy); 20.0% degenerative disc and protrusion; 13.9% disc herniation. Most common lumbar spine disc level affected included L5-S1 disc level (59.1%) and L4-L5 disc level (48.7%); more than one disc levels were affected in 45.2% of participants Fig. 2. Ligamentum flavum hypertrophy was recorded in 58.3% of study participants.

### Clinical characteristics of cases

The mean age of onset of symptoms of LSS was 50.3 ± 11.6 years; 49.3 ± 12.4 years for females and 51.4 ± 10.7 years for males (*p* = 0.488). Low back pain (100.0%), radicular symptoms (98.2%) and neurogenic claudication (98.2%) were the most common presenting symptoms. Low back pain was described as cramping by 77.2% of cases and 57.9% of cases said the timing of pain was intermittent; with 8.8% of cases reporting nocturnal symptoms. Cases who admitted having reduced walking distances constituted 98.2%, while cases who admitted having impaired routine daily activities constituted 96.5% Table 2.

**Table 2:** Clinical characteristics of cases

Parameter	Value
Age of onset (years), mean ± SD	50.3 ± 11.6
Low back pain, n (%)	57 (100)
Lower limb numbness, n (%)	48 (84.2)
Lower limb weakness, n (%)	47 (82.5)
Urinary incontinence, n (%)	1 (1.8)
Saddle anaesthesia, n (%)	1 (1.8)
Radiculopathy, n (%)	56 (98.2)
L4	13 (22.8)
L5	39 (68.4)
S1	4 (7.0)
Neurogenic claudication, n (%)	56 (98.2)
Nature of pain, n (%)	
Cramping	44 (77.2)
Burning	12 (21.1)
Ill defined	1 (1.8)
Aggravating factors, n (%)	
Walking long distances	56 (98.2)
Standing erect	56 (98.2)
Coughing	46 (80.7)
Defecation	38 (66.7)
Relieving Factor, n (%)	
Leaning forward/sitting/stooping	55 (96.5)
Timing, n (%)	
Intermittent	33 (57.9)
Nocturnal	5 (8.8)
^a^Pain Grading, mean ± SD	8.4 ± 1.1
Impact on daily activities, n (%)	
Reduced walking distance	56 (98.2)
Impaired routine daily activities	55 (96.5)
Physical examination, n (%)	
Altered gait	51 (89.5)
Lumbar tenderness	51 (89.5)
^b^Motor deficits	13 (22.8)
Abnormal (reduced) reflexes	20 (35.1)
Positive straight leg raise test	41 (71.9)

^a^Pain grading on VAS

^b^Motor power < 5

### Factors associated with presence of symptoms of LSS in patients with radiographic LSS

On bivariate analysis, clinical features of a positive family history of chronic low back pain (OR: 5.39, *p* < 0.001), grand multi-parity (OR: 3.50, *p* = 0.018), obesity (OR: 18.20, *p* < 0.001), and high waist circumference (OR: 2.44, *p* = 0.002) were significantly associated with presence of LSS symptoms in persons with radiographic LSS. Radiographic features of vertebra lesion at L3 level (OR: 3.11, *p* = 0.033), degenerative disc protrusion (OR: 4.13, *p* = 0.015), degenerative disc herniation (OR: 4.84, *p* = 0.017), L3-L4 disc lesion (OR: 8.49, *p* < 0.001), L4-L5 disc lesion (OR: 3.80, *p* = 0.001) and multi-level disc lesion (OR: 3.29, *p* = 0.002) were also significantly associated with presence of LSS symptoms in persons with radiographic LSS. However, age (OR: 1.02, *p* = 0.130), sitting height (OR: 0.98, *p* = 0.511), relative sitting height (OR: 1.05, *p* = 0.434), type of radiographic stenosis (*p* = 0.991), type of vertebra lesion (*p* = 0.261), type of disc lesion (*p* = 0.197) and ligamentum flavum hypertrophy (OR: 0.84, *p* = 0.648) were not significantly associated with presence of symptoms of LSS in these persons.

On multivariate analysis adjusted for BMI class, a positive family history of low back pain (OR: 3.89, *p* = 0.004), vertebra lesion at L2 (OR: 5.30, *p* = 0.034), L3 (OR: 6.73, *p* = 0.002), L4 (OR: 2.98, *p* = 0.025) and multiple (OR: 3.80, *p* = 0.008) levels, degenerative disc protrusion (OR: 4.08, *p* = 0.044), disc lesion at L3-L4 (OR: 9.44, *p* = 0.001), L4-L5 (OR: 3.33, *p* = 0.011) and multiple (OR: 2.54, *p* = 0.046) levels were significantly associated with presence of symptoms of LSS in persons with radiographic LSS Table 3.

**Table 3:** Factors associated with presence of symptoms of LSS in patients with radiographic LSS

Parameter	^a^OR (95% CI)	*p value*	^b^aOR	*p value*
Age	1.023 (0.993–1.052)	0.130	1.006 (0.972–1.041)	0.732
Gender (Females)	0.969 (0.465–2.019)	0.932	0.508 (0.199–1.295)	0.156
Family history of CLBP	5.388 (2.404–12.076)	<0.001	3.885 (1.531–9.858)	0.004
Grand multiparity	3.500 (1.238–9.891)	0.018	1.596 (0.435–5.861)	0.481
BMI Class				
Normal	Ref.	–	–	–
Overweight	1.156 (0.426–3.132)	0.776	–	–
Obese	18.200 (5.551–59.670)	<0.001	–	–
High waist circumference	2.442 (1.151–5.182)	0.020	–	–
Sitting height	0.981 (0.925–1.039)	0.511	1.057 (0.977–1.144)	0.165
Relative sitting height	1.049 (0.130–1.184)	0.434	1.087 (0.934–1.266)	0.279
Radiographic Stenosis		0.991		0.956
Central	Ref.	–	Ref.	–
Foraminal	0.949 (0.450–2.001)	0.890	0.871 (0.352–2.153)	0.871
Lateral	–	0.999	–	0.999
Type of vertebra lesion		0.261		0.800
None	Ref.	–	Ref	–
IAJOH	1.692 (0.687–4.167)	0.253	1.404 (0.469–4.202)	0.544
Osteophytes	0.547 (0.181–1.658)	0.286	0.662 (0.177–2.485)	0.542
Listhesis	–	0.999	–	0.999
Tumoral	–	0.999	–	0.999
Vertebra lesion level				
L1	2.113 (0.371–12.021)	0.399	5.703 (0.959–33.911)	0.056
L2	1.910 (0.434–8.413)	0.392	5.298 (1.131–24.526)	0.034
L3	3.111 (1.097–8.825)	0.033	6.733 (2.024–22.398)	0.002
L4	1.902 (0.897–4.033)	0.094	2.980 (1.145–7.757)	0.025
L5	1.308 (0.563–3.035)	0.532	1.399 (0.494–3.961)	0.527
Multi-level	1.818 (0.865–3.821)	0.115	3.798 (1.408–10.246)	0.008
Type of disc lesion		0.197		0.509
None	Ref.	–	Ref.	–
IDD	1.980 (0.614–6.382)	0.253	2.933 (0.732–11.751)	0.129
IDP	1.833 (0.451–7.454)	0.397	1.596 (0.282–9.039)	0.597
IDH	2.200 (0.517–9.356)	0.286	3.433 (0.645–18.263)	0.148
HP	2.200 (0.270–17.924)	0.461	1.244 (0.086–17.912)	0.872
DDP	4.125 (1.322–12.872)	0.015	4.084 (1.041–16.021)	0.044
DDH	4.840 (1.326–17.666)	0.017	2.641 (0.532–13.115)	0.235
Disc lesion level				
L1-L2	–	0.999	–	0.999
L2-L3	7.980 (0.949–67.112)	0.056	9.122 (0.941–88.425)	0.056
L3-L4	8.486 (2.695–26.722)	<0.001	9.436 (2.497–35.653)	0.001
L4-L5	3.797 (1.754–8.221)	0.001	3.331 (1.312–8.457)	0.011
L5-S1	0.782 (0.371–1.648)	0.518	0.592 (0.240–1.462)	0.256
Multi-level	3.285 (1.524–7.079)	0.002	2.542 (1.016–6.361)	0.046
Ligamentum Flavum hypertrophy	0.841 (0.401–1.766)	0.648	0.708 (0.289–1.735)	0.450

*OR* odds ratio, *aOR* adjusted odds ratio, *CLBP* chronic low back pain, *IAJOH* inter apophysial joint osteoarthritis and hypertrophy, *IDD* isolated degenerative disc, *IDP* isolated disc protrusion, *IDH* Isolated disc herniation, *HP* herniation and protrusion, *DDP* degenerative disc and protrusion, *DDH* degenerative disc and herniation

^a^Odds ratio of having symptoms of Lumbar Spinal Stenosis on bivariate analysis

^b^adjusted odds ratio of having symptoms of Lumbar Spinal Stenosis on multivariate analysis, adjusted for BMI class

## Discussion

We found no clear association between radiographic features and presence of symptoms of LSS confirming the difficulty to predict occurrence of symptoms of LSS from radiographic findings [26, 47–49]. Obesity, high waist circumference, a positive family history of low back pain, vertebra lesion at L2, L3, L4 and multiple levels, degenerative disc protrusion, disc lesion at L2-L3, L3-L4, L4-L5 and multiple levels were significantly associated with occurrence of symptoms of LSS in persons with radiographic LSS.

The mean age of onset of symptoms of LSS was found in our study was similar to reports published on a Caucasian population [45], thus not supporting the assertion that the tough nature of tasks carried out by Africans makes them prone to develop symptomatic LSS at younger ages compared to Westerners [8, 50]. We found no significant difference between the mean ages of symptomatic and asymptomatic persons with radiographic LSS (*p* = 0.128), attesting that degenerative changes observed in LSS are part of the normal aging process [31, 51], and do not fully account for the presence of symptoms of LSS. Other associated factors may therefore account for the presence of LSS symptoms in persons with radiographic LSS.

In our study, symptoms of LSS were worsened by standing erect in 98.2% of cases, and relieved by leaning forward/sitting/stooping in 96.5% of cases, consistent with reports of an important dynamic component in LSS [22]. Hirasawa et al. reported changes in the mean dural sac antero-posterior diameter and cross-sectional area in response to the posture of asymptomatic volunteers [52]. The available space in the central canal and foramen decreases on loading and extension while it increases on axial distraction and flexion [9]. Patients therefore commonly adopt a position with hip and knee slightly flexed referred as ‘simian stance’ [53]. This is in conjunction with the Penning’s ‘rule of progressive narrowing’ which implies the narrower the canal by stenosis, the more it narrows with spinal extension [54]. The fact that postural changes, hence degree of narrowing, correlate with the severity of symptoms implies the size of the canal and foramen plays an important role in symptomatic LSS. Ischemia of nerve roots, resulting from neuro-vascular compression, leads to claudication pain in the muscles supplied by the nerve roots at the stenotic level [55].

Obesity and central obesity has been associated to the occurrence of symptoms in LSS as found in our study. Knutsson et al. [56] suggested obesity as a novel explanation for clinical LSS, but further research is needed to assess and explain this relationship. There has been a suggestion of a possible genetic component in clinical LSS [57], supported by findings of a significant association between a positive family history of chronic low back pain among first degree relatives and clinical LSS in our study. A qualitative and quantitative evaluation of lumbar MRI of male twins reported that LSS is highly genetic, and disc degeneration is one possible mechanism through which genes influence spinal stenosis [58].

Persons who had vertebra lesions at multiple levels and disc lesions at multiple levels had significant higher odds of having symptoms of LSS compared to persons who had at one level as found in other studies [29, 59], but contrasted findings by Lohman [25] who did not find any correlation between the number of stenotic levels and symptoms of LSS. Our finding could be explained by the fact that the anatomy of the venous supply of the roots of the cauda equina makes these roots only vulnerable to congestion at multiple levels [55]. A single low pressure block will only affect a small segment of the root and will probably not impair conduction, while multiple blocks will cause significant venous congestion and lead to claudication [55].

We found no clear association between radiographic and clinical LSS. The type of radiographic stenosis, type of vertebra lesion and type of disc disease were not significantly associated with presence of LSS symptoms in persons with radiographic LSS, confirming findings in other studies [24–30], though some contrasting findings have been reported [4]. LSS is not solely an anatomic condition and other associated factors are responsible for the occurrence of symptoms as explained by Porter [55]; a shallow lumbar canal is only one factor in the pathophysiology of clinical LSS.

The observed ambiguous correlation between radiographic and clinical LSS can be accounted by a number of reasons. There is no consensus on the diagnostic criteria for radiographic LSS [19]. Different parameters with different cut-off values are currently being used to define radiographic stenosis. Furthermore, the effect of growth, body height and body size on these parameters are not known [60, 61]. Also, different imaging tools are used to assess anatomic stenosis. MRI is widely accepted as the preferred tool because of its ability to clearly depict soft tissue [17]. CT scan is used in situations where MRI is not readily available, in persons with contraindications to MRI and for pre-surgical planning to depict bony structures [19]. In addition to these shortcomings of radiographic LSS, the clinical diagnosis of LSS and has certain limitations; the symptoms felt by patients are highly subjective and patients report these symptoms differently. Also, these symptoms are influenced by psychological factors such as depression [62] and anxiety [63]. These different factors could explain why persons with severe radiographic stenosis may present with little or no symptoms, while others with mild radiographic stenosis may present with severe disabling symptoms.

Our study had strengths and limitations. To the best of our knowledge, this is the first study in sub-Saharan Africa to determine factors associated with presence of symptoms of LSS in radiographic LSS. We explored a wide range of clinical and radiographic variables. However, we did not assess psychological factors such as depression and anxiety which may impact presence of symptoms in radiographic LSS [62, 63]. Despite the fact that MRI is widely used and is regarded as the best imaging tool to assess LSS [17], we used CT scan in this study because of easy accessibility in this resource limited setting. The study design did not allow us explore causal relationships between the associations observed.

## Conclusion

Characteristics of clinical LSS have been described in this sub-Saharan Africa population. Anatomic stenosis plays an important role in clinical LSS but is not solely accountable. Obesity, high waist circumference, a positive family history of low back pain, vertebra lesion at L2, L3, L4 and multiple levels, degenerative disc protrusion, disc lesion at L2-L3, L3-L4, L4-L5 and multiple levels are significantly associated with presence of symptoms of LSS in persons with radiographic LSS.

## Abbreviations

BMI: Body mass index
CT scan: Computed tomography scan
LSS: Lumbar spinal stenosis
MRI: Magnetic resonance imaging
VAS: Visual analogue scale
WC: Waist circumference

## References

[1] Arnoldi CC, Brodsky AE, Cauchoix J, Crok HV, Dommisse GF, Edgar MA, Gargano FP, Jacobson RE, Krikaldy-Willis WH, Kurihara A (1976). Lumbar spinal stenosis and nerve root entrapment syndromes: definition and classification. Clin Orthop Relat Res.

[2] Katz JN, Harris MB (2008). Lumbar spinal stenosis. N Engl J Med.

[3] Krikaldy-Willis WH, McIvor GW (1976). Editorial: Lumbar spinal stenosis. Clin Orthop Relat Res.

[4] Kalichman L, Cole R, Kim DH, Li L, Suri P, Guermazi A, Hunter DJ (2009). Spinal stenosis prevalence and association with symptoms: the Framingham study. Spine J.

[5] Hsiang JK, Furman MB. Epidemiology. In: Spinal Stenosis. Medscape. 2017. https://emedicine.medscape.com/article/1913265-overview#a6. Accessed 24 Nov 2017.

[6] Ishimoto Y, Yoshimura N, Muraki S, Yamada H, Nagata K, Hashizume H, Takiguchi N, Minamide A, Oka H, Kawaguchi H (2013). Associations between radiographic lumbar spinal stenosis and clinical symptoms in the general population: the Wakayama spine study. Osteoarthr Cartil.

[7] Kabre A, Ba MC, Cisse R, Sorgho Lougue C, Doli P, Kabore J (2003). Lumbar canal stenosis in Ouagadougou: aetiological, clinical aspects and prognosis regarding 80 cases. Dakar Med.

[8] Oniankitan O, Magnan A, Fianyo E, Mijiyawa M (2007). Lumbar canal stenosis in an outpatient clinic in Lome, Togo. Med Trop.

[9] Schönström N, Lindhal S, Willén J, Hansson T (1989). Dynamic changes in the dimensions of the lumbar spinal canal: an experimental study in vitro. J Orthop Res.

[10] Alvarez JA, Hardy RH (1998). Lumbar spine stenosis: a common cause of back and leg pain. Am Fam Physician.

[11] Naghavi N, Wang H, Lozano R, Davis A, Liang X, Zhou M, Vollst SE, Ozgoren AA, Abdalla S, Abd-Allah F (2013). Global, regional, and national age-sex specific all-cause and cause-specific mortality for 240 causes of death, 1990–2013;2013: a systematic analysis for the global burden of disease study 2013. Lancet.

[12] United Nations. World population ageing 2013. Department of Economic and Social Affairs Population Division 2013.

[13] Deyo RA, Mirza SK, Martin BI, Kreuter W, Goodman DC, Jarvik JG (2010). Trends, major medical complications and charges associated with surgery for lumbar spinal stenosis in older adults. JAMA.

[14] Stromqvist B, Fritzell P, Hagg O, Jonsson B (2009). The swedish spine register: development, design and utility. Eur Spine J.

[15] Suri P, Rainville J, Kalichman L, Katz JN (2010). Does this older adult with lower extremity pain have the clinical syndrome of lumbar spinal stenosis?. JAMA.

[16] Steurer J, Roner S, Gnannt R, Hodler J (2011). Quantitative radiologic criteria for the diagnosis of lumbar spinal stenosis: a systematic literature review. BMC Musculoskelet Disord.

[17] Malfair D, Beall DP (2007). Imaging the degenerative diseases of the lumbar spine. Magn Reson Imaging Clin N Am.

[18] Genevay S, Atlas SJ, Katz JN (2010). Variation in eligibility criteria from studies of radiculopathy due to a herniated disc and of neurogenic claudication due to lumbar spinal stenosis: a structured literature review. Spine (Phila Pa 1976).

[19] Andreisek G, Hodler J, Steurer J (2011). Uncertainties in the diagnosis of lumbar spinal stenosis. Radiology.

[20] Verbiest H (1975). Pathomorphologic aspects of developmental lumbar stenosis. Orthop Clin North Am.

[21] Ishimoto Y, Yoshimura N, Muraki S, Yamada H, Nagata K, Hashizume H, Takiguchi N, Minamide A, Oka H, Kawaguchi H (2012). Prevalence of symptomatic lumbar spinal stenosis and its association with physical performance in a population-based cohort in Japan: the Wakayama spine study. Osteoarthr Cartil.

[22] Genevay S, Atlas SJ (2010). Lumbar spinal stenosis. Best Pract Res Clin Rheumatol.

[23] Hurri H, Slätis P, Soini J, Tallroth K, Alaranta H, Laine T, Heliövaara M (1998). Lumbar spinal stenosis: assessment of long-term outcome 12 years after operative and conservative treatment. J Spinal Disord.

[24] Amundsen T, Weber H, Lilleås F, Nordal HJ, Abdelnoor M, Magnaes B (1995). Lumbar spinal stenosis. Clinical and radiologic features. Spine.

[25] Lohman CM, Tallroth K, Kettunen JA, Lindgren K-A (2006). Comparison of radiologic signs and clinical symptoms of spinal stenosis. Spine.

[26] Geisser ME, Haig AJ, Tong HC, Yamakawa KS, Quint DJ, Hoff JT, Miner JA, Phalke JJ (2007). Spinal canal size and clinical symptoms among persons diagnosed with lumbar spinal stenosis. Clin J Pain.

[27] Jonsson B, Annertz M, Sjoberg C, Stromqvist B (1997). A Prospective and consecutive study of surgically treated lumbar spinal stenosis. Part I: clinical features related to radiographic findings. Spine (Phila Pa 1976).

[28] Sirvanci M, Bhatia M, Ganiyusufoglu KA, Duran C, Tezer M, Ozturk C, Aydogan M, Hamzaoglu A (2008). Degenerative lumbar spinal stenosis: correlation with Oswestry disability index and MR imaging. Eur. Spine J.

[29] Hong JH, Lee MY, Jung SW, Lee SY (2015). Does spinal stenosis correlate with MRI findings and pain, psychologic factor and quality of life?. Korean J Anesthesiol.

[30] Kuittinen P, Sipola P, Saari T, Aalto TJ, Sinikallio S, Savolainen S, Kröger H, Turunen V, Leinonen V, Airaksinen O (2014). Visually assessed severity of lumbar spinal canal stenosis is paradoxically associated with leg pain and objective walking ability. BMC Musculoskelet Disord.

[31] Boden SD, Davis DO, Dina TS, Patronas NJ, Wiesel SW (1990). Abnormal magnetic-resonance scans of the lumbar spine in asymptomatic subjects. A prospective investigation. J Bone Joint Surg Am.

[32] Katz JN, Lipson SJ, Chang LC, Levine SA, Fossel AH, Liang MH (1996). Seven- to 10-year outcome of decompressive surgery for degenerative lumbar spinal stenosis. Spine.

[33] Atlas SJ, Keller RB, YA W, Deyo RA, Singer DE (2005). Long-term outcomes of surgical and nonsurgical management of lumbar spinal stenosis: 8–10 year results from the Maine lumbar spine study. Spine.

[34] International standard classification of occupations (2007). International Labour Organization.

[35] Price DD, McGrath PA, Rafii A, Buckingham B (1983). The validation of visual analogue scales as ratio scale measures for chronic and experimental pain. Pain.

[36] World Health Organization (2011). Waist circumference and waist-hip ratio: Report of a WHO expert consultation, Geneva, 8–11 December 2008.

[37] Carr RV, Rempel RD, Ross WD (1989). Sitting height: an analysis of five measurement techniques. Am J Phys Anthropol.

[38] Bardeen CR (1923). General relations of sitting height to stature and of sitting height and stature to weight. American Journal of Physical Anthropolgy.

[39] Lee B, Kazam E, Newman A (1978). Computed tomography of the spine and spinal cord. Radiology.

[40] Mamisch N, Brumann M, Hodler J, Held U, Brunner F, Steurer J (2012). Radiologic criteria for the diagnosis of spinal stenosis: results of a Delphi survey. Radiology.

[41] Ullrich C, Binet E, Sanecki M, Kieffer S (1980). Quantitative assessment of the lumbar spinal canal by computed tomography. Radiology.

[42] Schonstrom N, Bolender N, Spengler D (1985). The pathomorphology of spinal stenosis as seen on CT scans of lumbar spine. Spine.

[43] Mikhael MA, Ciric I, Tarkington JA (1981). Neuroradiological evaluation of lateral recess syndrome. Radiology.

[44] Ciric I, Mikhael MA, Tarkington JA (1980). The lateral recess syndrome. A variant of spinal stenosis. Neurosurgery.

[45] Strojnik T (2001). Measurement of the lateral recess angle as a possible alternative for evaluation of the lateral recess stenosis on CT scan. Wein. Klin Wochenschr.

[46] Beers BJ, Carter AP, Leiter BE, Tilak SP, Shah RR (1985). Interobserver discrepancies in distance measurements from lumbar spine CT scans. AJR Am J Roentgenol.

[47] Borenstein DG, O'Mara JW, Boden SD, Lauerman WC, Jacobson A, Platenberg C, Schellinger D, Wiesel SW (2001). The value of magnetic resonance imaging of the lumbar spine to predict low-back pain in asymptomatic subjects: a seven-year follow-up study. J Bone Joint Surg Am.

[48] Maus T (2010). Imaging the back pain patient. Phys Med Rehabil Clin N Am.

[49] Haig AJ, Geisser ME, Tong HC, Yamakawa KS, Quint DJ, Hoff JT, Chiodo A, Miner JA, Phalke VV (2007). Electromyographic and magnetic resonance imaging to predict lumbar stenosis, low-back pain, and no back symptoms. J Bone Joint Surg Am.

[50] Getty C (1980). Lumbar spinal stenosis: the clinical spectrum and the results of operation. J Bone Joint Surg Br.

[51] Brinjikji W, Leutmer PH, Comstock B, Bresnaham BW, Chen LE, Deyo RA, Halabi S, Turner JA, Avins AL, James K (2015). Systematic literature review of imaging features of spinal degeneration in asymptomatic populations. AJNR Am J Neuroradiol.

[52] Hirasawa Y, Bashir WA, Smith FW, Magnusson ML, Pope MH, Takahashi K (2007). Postural changes of the dural sac in the lumbar spines of asymptomatic individuals using positional stand-up magnetic resonance imaging. Spine (Phila Pa 1976).

[53] Bridwell KH (1994). Lumbar spinal stenosis. Diagnosis, management, and treatment. Clin Geriatr Med.

[54] Penning L (1992). Functional pathology of lumbar spinal stenosis. Clin Biomech.

[55] Porter RW (1996). Spinal stenosis and neurogenic claudication. Spine.

[56] Knutsson B, Sandén B, Sjödén G, Järvholm B, Michaëlsson K (2015). Body mass index and risk for clinical lumbar spinal stenosis: a cohort study. Spine (Phila Pa 1976).

[57] Noponen-Hietala N, Kyllönen E, Männikkö M, Ilkko E, Karppinen J, Ott J, Ala-Kokko L (2003). Sequence variations in the collagen IX and XI genes are associated with degenerative lumbar spinal stenosis. Ann Rheum Dis.

[58] Battié MC, Ortega-Alonso A, Niemalainen R, Gill K, Levalahti E, Videman T, Kaprio J (2014). Lumbar spinal stenosis is a highly genetic condition partly mediated by disc degeneration. Athritis Rheumatol.

[59] Porter RW, Ward D (1992). Cauda equina dysfunction. The significance of two-level pathology. Spine.

[60] Knirsch W, Kurtz C, Häffner N, Langer M, Kececioglu D (2005). Normal values of the sagittal diameter of the lumbar spine (vertebral body and dural sac) in children measured by MRI. Pediatr Radiol.

[61] Dora C, Wälchli B, Elfering A, Gal I, Weishaupt D, Boos N (2002). The significance of spinal canal dimensions in discriminating symptomatic from asymptomatic disc herniations. Eur. Spine J.

[62] McKillop AB, Carroll LJ, Battié MC (2014). Depression as a prognostic factor of lumbar spinal stenosis: a systematic review. Spine J.

[63] Athiviraham A, Wali ZA, Yen D (2011). Predictive factors influencing clinical outcome with operative management of lumbar spinal stenosis. Spine J.

